# Between surplus and support: a comparison between Greater Manchester (UK) and Kyoto Prefecture (Japan) charitable food provision fields

**DOI:** 10.1007/s41130-025-00241-4

**Published:** 2025-11-06

**Authors:** Filippo Oncini, Hein Mallee

**Affiliations:** 1 Maastricht Sustainability Institute, Maastricht University, Tapijnkazerne 11, 6211ME Maastricht, Netherlands; 2 Department of Japanese Food Culture, Kyoto Prefectural University, Sakyo-ku, 1-5 Shimogamo Hangi-cho, Kyoto (606-8522) Japan

**Keywords:** Field theory, Food banks, Food charity, Food insecurity, Surplus food, Sustainability

## Abstract

Over the past decades, charitable food provision (CFP) has become a vital safety net in addressing poverty across the Global North. Organizations distributing food parcels or meals, often sourced from surplus, now play a central role in urban poverty relief. Framed as a “win–win” solution that aligns social and environmental sustainability, CFP is widely perceived as capable of addressing hunger while reducing food waste. This paper offers an innovative, field-level comparison of CFP systems in Greater Manchester (UK) and Kyoto Prefecture (Japan) using Strategic Action Field (SAF) theory—a framework not yet widely applied in this context. We analyze the emergence of these fields, their operational dynamics, interactions with state and corporate actors, and the impact of COVID-19. Drawing on extensive qualitative data, including semi-structured interviews and field observations, we identify contrasting organizational models: the UK’s formalized, network-driven approach versus Japan’s decentralized, community-oriented one. Despite these differences, both fields depend on volunteers, corporate partnerships, and expanded activity during the pandemic. By showing how different institutional logics shape the articulation of social and environmental sustainability, the paper not only critiques the “win–win” narrative but also advances a comparative framework for future research into CFP fields.

## Introduction

One of the reasons behind the growth and success of charitable food provision (CFP) throughout the Global North lies in the win–win “narrative plot” (Ariztia & Araneda, 2022) that underpins much of the public discourse on the topic (Arcuri, 2019; Caplan, 2017). A “win–win” situation refers to a scenario in which all parties involved benefit or achieve favorable outcomes, without a clear “loser.” Following this logic, the functional complementarity between food waste prevention and food insecurity alleviation is particularly appealing, both morally and pragmatically, as it suggests that their synergy produces a net advantage for both the environment and society by reducing greenhouse gas emissions while sustaining people’s food needs (Galli et al., 2019). This dual benefit frames CFP not only as an efficient solution but also as a responsible endeavor, connecting different Sustainable Development Goals (particularly 1, 2 and 12)^1^ in a way that appeals to a broad spectrum of stakeholders, seemingly aligning social and environmental sustainability goals.

Academic literature has criticized such framing for decades (Poppendieck, 1994, 1999; Riches, 2018). As a system for food support distribution, CFP is often characterized by operational challenges, including inadequate food provision, supply instability, and the stigma and shame experienced by recipients (Bazerghi et al., 2016; Garthwaite, 2016; McIntyre et al., 2016; Middleton et al., 2018). Concurrently, corporate actors, particularly in the food industry, engage with food charities for cosmetic purposes, if not outright greenwashing (Fisher, 2017; Lambie-Mumford & Kennedy, 2025). By leveraging their donations and partnerships with food charities, manufacturers, retailers, and producers can improve their public image while diverting attention from systemic issues like food insecurity, waste, and labor exploitation, sometimes even embedded in their own practices (Caplan, 2017). Moreover, this approach potentially discourages critical reflection on the overproduction that drives food waste, as it frames excess production as a charitable resource rather than a sign of inefficiency within the food system.

While it is undeniable that these trends and criticisms apply broadly across contexts, there remains a notable gap in comparative research examining how these dynamics manifest in different socio-political environments. A comparative lens is essential because it can illuminate how distinct historical, cultural, and policy contexts shape the evolution and operation of CFP, as well as the different articulation of its goals. Thus, CFP serves as an ideal phenomenon for exploring the tensions, contradictions, and articulations of social and environmental sustainability. Through comparison, we can uncover the different ways in which they are defined and prioritized in diverse settings, as well as the unique challenges and opportunities that arise in each context. Hence, this paper asks how do CFP fields differ in their emergence, organization, and engagement with social and environmental sustainability, and what do these differences reveal about the institutional logics underpinning each system?

To respond to this question, we build on two in-depth case studies conducted in Greater Manchester (UK) and Kyoto Prefecture (Japan) over recent years to delineate similarities and differences in the organization of CFP across these two markedly different contexts. These cases were selected for both theoretical and pragmatic reasons. Analytically, they offer contrasting welfare regimes and civil society traditions, providing valuable leverage to investigate how CFP fields emerge and operate under different socio-political conditions. The UK exemplifies a liberal welfare state with a highly formalized and networked CFP field, while Japan’s statist welfare approach, typical of the East Asian model, relies strategically on non-state actors, supporting a decentralized and community-driven field. Pragmatically, the selection was supported by the availability of research funding and institutional affiliations in both countries, which enabled extensive fieldwork and sustained engagement with key actors and organizations. More broadly, the comparison underscores the value of examining CFP fields across different socio-economic and cultural milieus across high-income countries, moving beyond European or US perspectives.

After introducing key concepts related to social and environmental sustainability and illustrating their relevance in the context of food insecurity and surplus food distribution, we approach the comparison by framing both CFP systems as fields, specifically as strategic action fields (SAFs) (Fligstein & McAdam, 2012; Oncini, 2023, 2024; Oncini & Ciordia, 2024). Using the SAF framework, we analyze how these fields are structured and function, focusing on several key dimensions. We highlight key differences and similarities concerning (i) the emergence of the CFP fields in both countries, investigating in particular the different role played by surplus food distribution; (ii) the shared understandings and practices of the actors involved, exploring how these actors perceive and engage with food provision; (iii) the relationships between organizations within each field and the broader environment, particularly their interaction with the state and corporate partners; and (iv) the impact of COVID-19 as an external shock, assessing how the pandemic has influenced and reshaped the dynamics and operations of CFP in each context. For each of these blocks, we disentangle differences in how social and environmental sustainability relate in each context, distinguishing between *framing* (how they are conceptualized), *application* (how they are implemented), and *exploitation* (how they are utilized in order to benefit from them). Finally, the discussion section outlines the limitations and advantages of this approach and further reflects on the possibility of extending the comparison of CFP fields to other contexts within and between countries.

## Charitable food provision: environmental or social sustainability?

The concept of sustainability has evolved significantly over time. Originally, sustainability was closely tied to ecological and environmental concerns, focusing on maintaining the capacity of natural systems to support life and ensuring that resources were used in ways that allowed future generations to thrive. Still in 1998, Hueting and Reijnders (1998, p. 143) argued in their paper “Sustainability is an Objective Concept”: “Although historians of future generations may most certainly be interested in the social constructs we devised with respect to physical reality, the generations in question will be confronted primarily with reality and not with the contemporary social construct we made of it. There is thus every reason to reject the idea that sustainability is a subjective concept or a contemporary social construct.”

This definition of sustainability limits its scope to the environmental dimension, often conceptualized through the framework of “planetary boundaries.” These boundaries represent critical thresholds within Earth’s biophysical systems—such as climate regulation, biodiversity, land use, and freshwater cycles—that must not be exceeded to ensure a safe operating space for humanity (Rockström et al., 2009). Significant differences exist in how these boundaries are assessed or prioritized, and new boundaries are discovered and added with time (Richardson et al., 2023). Nevertheless, there is broad scientific consensus on the fundamental actions required to maintain ecological balance and mitigate climate change, such as transitioning away from fossil fuels, promoting afforestation, and adopting sustainable land-use practices to restore ecosystems and enhance carbon sequestration; all these measures are critical to reducing greenhouse gas emissions, which are the main drivers of climate change (IPCC, 2022). In food systems, a critical example is food waste prevention. Estimates indicate that food loss and waste account for roughly 8% to 10% of global greenhouse gas emissions (FAO, 2013), primarily through the release of carbon dioxide (CO₂) and methane (CH₄).

From an environmental sustainability perspective, preventing food waste is an essential strategy for mitigating climate change. However, the challenge lies not only in identifying what must be done but in determining how to achieve it. Any meaningful sustainability transition must consider the social systems and institutions that shape these processes.^2^ This interdependence was central to the broader understanding of sustainability introduced in the Brundtland Report (WCED, 1987), which emphasized the interconnectedness of environmental, social, and economic dimensions under the framework of “sustainable development.” Decisions on reducing food waste—or more broadly, addressing the negative externalities of the food system—are inherently tied to social structures, tying environmental, social and economic sustainability together. However, while the environmental and economic pillars of sustainability often rely on measurable outcomes and specific targets, social sustainability is sufficiently appealing and vague to attract widespread support without necessarily requiring consensus on concrete actions or measurable outcomes (Boström, 2012).^3^ In fact, depending on the definitions, social sustainability can be seen as a stand-alone, macro-level objective aiming to ensure the well-being of communities and individuals, or rather as a place-based and process-oriented framework guiding participatory governance, local engagement, and integrated solutions that account for diverse perspectives and cultural contexts (Boyer et al., 2016).

Vallance et al.’s (2011) distinction between three forms of social sustainability—development, bridge, and maintenance sustainability—is useful to unpack the concept and to better investigate the relevant ambiguities within CFP. On the surface, all three dimensions share an alignment with the fundamental goal of addressing food insecurity and fostering a more equitable and sustainable society. Development sustainability, focusing on addressing basic needs, equity, and reducing deprivation, resonates with CFP’s mission to meet immediate needs and reduce deprivation by redistributing surplus food to vulnerable populations. Bridge sustainability, namely the change in social practices so as to achieve environmental goals, clearly connects with the ecological benefits of CFP, as repurposing surplus food helps reduce waste and greenhouse gas emissions. Finally, maintenance sustainability, defined as the preservation of cultural and social practices valued by communities, can connect to CFP in two ways. On the one hand, it aligns with historical and religious traditions of giving alms to the poor, reinforcing long-standing practices of care and solidarity within communities (Carstairs, 2017; Goodwin, 1994). On the other hand, maintenance sustainability also draws on the moral and political urgency surrounding food waste, mobilizing contemporary ethical and environmental concerns to justify charitable redistribution, even as it reinforces existing structures of surplus management (e.g., Lohnes, 2021).

However, while these dimensions align in principle, they often come into conflict when applied in practice (Vallance et al., 2011). For example, development sustainability, which focuses on addressing immediate food insecurity, often clashes with bridge sustainability’s environmental objectives. CFP acts as a buffer for both problems—feeding the hungry and reducing food waste—but it does not aim to resolve the root causes of either. By relying on surplus food, it sustains systems of overproduction and waste, undermining efforts to transform food systems into models that align with long-term ecological balance. Equally, tensions emerge between development and maintenance sustainability. While CFP addresses urgent needs by redistributing surplus food, it restricts recipients’ freedom to choose the food they consume (Moraes et al., 2021; Oncini, 2023). This lack of agency can conflict with cultural norms and traditions, particularly since food consumption and avoidance hold significant social or symbolic meanings (Fielding-Singh, 2017; Oncini et al., 2023). What people need to survive in the short term can inadvertently clash with their desire to uphold cultural practices, highlighting a conflict between meeting basic needs and respecting social norms.

At the core of these contradictions lies what Darmon (2024) describes as the inequality-unsustainability nexus, where systemic inequalities and environmental degradation reinforce one another under the capitalist growth imperative. This nexus is starkly evident in CFP, which alleviates food insecurity while simultaneously sustaining the systems of overproduction and waste that underpin capitalist food systems. By framing surplus redistribution as a win–win solution, CFP mitigates symptoms of inequality and unsustainability while leaving their structural roots unchallenged. Crucially, this dynamic rests on the implicit assumption that the third sector, embodied by charitable food organizations, should remain apolitical, focusing on short-term relief rather than systemic transformation. This depoliticization shields CFP from critical scrutiny, allowing it to operate within and perpetuate existing power structures rather than contesting them.

## Charitable food provision as a strategic action field

Over the past decades, field theories have been particularly helpful in examining the relational dynamics that shape social action within bounded arenas of shared meaning and structure (Barman, 2016). Field theories provide an alternative to reductionist micro-level approaches focused on individual behavior or macro-level systemic theories emphasizing overarching institutions. Instead, fields should be understood as meso-level social orders, where actors engage with one another based on shared understandings and power dynamics (Martin, 2003). Among the various field theories, strategic action field (SAF) theory, building on elements of Bourdieusian and New Institutionalism approaches, has emerged as a compelling framework for analyzing collective action and field-level dynamics (Fligstein & McAdam, 2012).

According to Fligstein and McAdam (2012), a SAF is “a constructed mesolevel social order in which actors (who can be individual or collective) are attuned to and interact with one another on the basis of shared (which is not to say consensual) understandings about the purposes of the field, relationships to others in the field (including who has power and why), and the rules governing legitimate action in the field” (Fligstein & McAdam, 2012: 9). SAF theory emphasizes both the structured and dynamic nature of fields, highlighting how cooperation and competition coexist and how external shocks or internal contestation can lead to significant transformations within the field.

Recently, SAF theory has been applied to the study of CFP to investigate how instances of cooperation and competition coexist, shaping the relationships between field members—namely, food charities—and their interactions with the broader field environment (Oncini, 2024). This environment consists of state and non-state actors that provide opportunities and impose constraints on food charities, thereby influencing both their operations and the experiences of the individuals they serve. SAF theory provides a valuable framework for examining not only the internal inertia within these organizations but also the complex relationships that structure the field order and drive its evolution. It enables an understanding of how stability is maintained while accounting for the forces that push toward change.

For the purpose of this paper, we employ four key building blocks of SAF theory to compare CFP in the UK and Japan and to examine how social and environmental sustainability are utilized and interpreted in these distinct contexts. First, by focusing on the CFP incumbents of both fields (i.e., the dominant actors in the arena), we illustrate the emergence and evolution of CFP fields in each country, showing how different path dependencies concerning the role of surplus food distribution have shaped the structure of the field and influenced the sustainability framing. Second, we focus on the shared understandings and practices among food charities, illustrating how operational methods converge or diverge between the two contexts, and then pointing out how sustainability is put into practice. Third, we turn to the broader field environment, focusing on the role of state actors and corporate partners, with attention to the ways sustainability is actually put into use. Finally, we examine the impact of the COVID-19 pandemic as an external shock to the field in both contexts that reinforced the current CFP field order and sustainability dynamics. COVID-19 constituted a major shared rupture that simultaneously impacted both the UK and Japan, providing a unique comparative lens through which to examine how existing CFP field structures and sustainability dynamics were either reinforced or contested.

## Data and methods

This study draws on two separate research projects conducted in Greater Manchester, UK, between 2019 and 2021, and Kyoto Prefecture, Japan, between 2022 and 2023. Guided by the SAF framework, both case studies explore the dynamics of CFP in the two city-regions by using semi-structured interviews, observations, and supplementary document analysis. In each context, participants were selected to maximize heterogeneity across CFP fields. The aim was to include diverse models of provision, such as meal providers, pantries, hybrid organizations so as to obtain a wider picture of how different organizational forms operate, interact, and respond to food insecurity within each field.

### Study contexts

National-level data from the Food and Agriculture Organization (FAO) indicate that moderate or severe food insecurity affected on average 5.7% of the UK population and 5.5% of the Japanese population between 2021 and 2023 (FAO et al., 2024). In both countries, major organizations reported that food support initiatives and requests for food assistance have consistently increased over the past decades (Kimura, 2018; The Trussell Trust, 2025). While city-level estimates are not available, these figures offer a useful backdrop for comparing the local dynamics of charitable food provision (CFP) in Greater Manchester and Kyoto Prefecture.

Greater Manchester (GM) is a metropolitan region in northwest England comprising ten local authority districts and a population of 2.8 million. Despite its transition from an industrial economy to a knowledge-based one, GM experiences significant economic disparities, with higher unemployment rates, lower average earnings, and notable levels of deprivation (Hughes & Lupton, 2017). The region hosts a dense network of food charities, ranging from small organizations with a single distribution center to national-level food charities like the Trussell Trust and FareShare.

Kyoto Prefecture, situated in the Kansai region of Japan, is home to approximately 2.5 million residents. Like the rest of Japan, Kyoto faces significant challenges stemming from prolonged economic stagnation and increasing precarity, which exacerbate economic uncertainties. These issues disproportionately affect vulnerable groups, such as single mothers and the elderly (Inaba, 2011; Raymo, 2022). Currently, nearly 20% of Kyoto’s population lives in relative poverty (Kubo, 2024), and not dissimilarly from GM, CFP in Kyoto is characterized by a mix of formal food banks, informal community-based initiatives, and several *kodomo shokudō* (children’s cafeterias),^4^ which provide free or low-cost meals and distribute food parcels to low-income families.

### Data collection and analysis

Table 1 summarizes the data collection strategy in both countries. Fieldwork in Greater Manchester took place between 2019 and 2021 and began with mapping the region’s food support organizations using resources from Greater Manchester Poverty Action (GMPA).^5^ This mapping exercise identified key types of food charities: food banks, pantries, and meal providers. A survey conducted with food charity directors captured operational changes during the early months of the COVID-19 pandemic (Oncini, 2022). This survey was followed by 30 semi-structured interviews with directors of food charities, stakeholders, and experts in surplus food redistribution. Additionally, 12 interviews were conducted with stakeholders such as advocacy group members and representatives of umbrella organizations like the Independent Food Aid Network (IFAN) and FareShare.

In Kyoto Prefecture, data was collected between 2022 and 2023, utilizing a combination of online research and snowball sampling to identify active food charities. This approach was necessitated by the absence of comprehensive mapping resources comparable to GMPA. A total of 19 semi-structured interviews were conducted with representatives from 14 food charities, alongside one interview with a civil servant with experience in CFP. These interviews were supplemented by observations at 8 food charity events, including food parcel assemblies and meal distribution activities. The observed initiatives included food banks, meal providers, *kodomo shokudō* and pantries, offering a diverse representation of CFP operations in Kyoto—and more generally, in Japan.

While the two fieldwork periods do not overlap, both took place during distinct phases of the COVID-19 pandemic. In Greater Manchester, data collection occurred during the first 2 years of the crisis, capturing both immediate responses and medium-term adaptations. In Kyoto, fieldwork took place after initial pandemic waves had subsided, but several distancing measures remained in place. Interview questions in Japan explicitly asked participants to reflect on how the early stages of the pandemic had affected their operations, allowing us to capture comparable insights across time and context. In both countries, the interviews focused on SAF-related themes, such as field rules, power structures, relationships within the field, and interactions between food charities and key state and non-state actors. They also explored differences in organizational practices and the impact of COVID-19 as an external shock. All interviews were recorded, anonymized, and transcribed.

**Table 1:** Table 1: Overview of data collection and research design

Category	Greater Manchester (UK)	Kyoto Prefecture (Japan)
Research period	2019–2021	2022–2023
Mapping strategy	Formal mapping using data from Greater Manchester Poverty Action (GMPA)	Informal mapping via online search and snowball sampling
Types of organizations studied	Food banks, pantries, meal providers, mixed, stakeholders	Food banks, meal providers, *kodomo shokudō*, stakeholders, pantries
Sampling strategy	Purposeful sampling; snowballing used for stakeholder interviews	Snowball sampling; X (formerly Twitter), use of community flyers and networks to reach informal groups
Number of interviews	42 total: 30 with food charity staff, 12 with stakeholders	20 total: 19 with food charity staff, 1 with a civil servant
Observations	Not conducted due to COVID-19 restrictions	8 events observed (e.g., food parcel assembly, meal distribution, *meal preparation*)
Supplementary data sources	GMPA data, charity reports, media articles, social media content	Leaflets, posters, internal materials from community events
COVID-19 component	Survey on early pandemic response; interviews explored crisis as field shock	Interviewees discussed pandemic impact and field adaptation
Interview handling	Semi-structured; all interviews recorded, anonymized, and transcribed	Semi-structured; all interviews recorded, anonymized, and transcribed
Limitations	No participant observation	Informal initiatives harder to trace; limited centralized data resources

The analysis proceeded iteratively, with coding guided by SAF theoretical blocks. This process included identifying field rules and shared understandings, mapping power structures and relational dynamics, and examining interactions between CFP fields and their broader environments. Comparative analysis emphasized differences in the emergence, evolution, and framing of social and environmental sustainability within each context. Supplementary data sources enriched the analysis. In GM, charity reports, social media posts, and media articles provided insights into public narratives and organizational strategies. In Kyoto, event observations and community-based documentation (e.g., leaflets and posters) highlighted the grassroots nature of many initiatives, offering a contrast to the more formalized operations in GM.

Ethical approval for the research was obtained from The University of Manchester and Kyoto Prefectural University, and all participants provided informed consent. Interviews were anonymized, and findings are presented in a way that protects participant confidentiality. Observations were conducted with the knowledge and consent of the organizations involved, ensuring transparency and respect for participants’ privacy.

In the analysis that follows, direct quotes from interviews are not included. Instead, the focus is placed on identifying and analyzing the broader similarities and differences in the functioning of CFP fields in Greater Manchester and Kyoto, with particular attention to their emergence and operational dynamics. This approach prioritizes the exploration of systemic patterns over individual or anecdotal evidence, allowing for a more comprehensive understanding of how the Strategic Action Field (SAF) framework serves as a functional comparative lens. This lens not only permits us to clearly identify the similarities and differences between the two CFP fields but also provides valuable insights into the intersection of social and environmental sustainability.

## Findings

### Emergence and current state of the field

In both the UK and Japan, CFP has expanded significantly in recent decades, emerging as a critical safety net. While the rise of organized food support systems in both countries reflects broader neoliberal transformations (e.g., Kimura, 2018; Möller, 2021), the trajectory and present-day set-up differ markedly. Understanding these differences is essential to grasp how CFP fields are currently organized and how social and environmental sustainability are framed in each context.

Historically, food support initiatives in both countries were rooted in the charitable efforts of religious institutions (Carstairs, 2017; Goodwin, 1994). However, the operational models of incumbent food charities and their divergent starting points have had significant implications for the evolution of CFP fields. These differences are particularly evident in the contrasting roles of surplus food redistribution and poverty alleviation within the two systems.

In the UK, Victorian welfare practices set the stage for many elements still visible in modern food charities. As Williams and May (2022) illustrate, organizations like the Charity Organization Society (COS), founded in 1869, emphasized the rationalization of almsgiving, including strict referral systems and the distribution of food tickets to prevent perceived misuse. Victorian-era soup kitchens, food tickets, and donation appeals incorporated paternalistic attitudes toward poverty, drawing clear distinctions between the “deserving” and “undeserving” poor (see also Garthwaite, 2016). At the same time, charitable food provision was already shaped by frugal logics that foreshadowed surplus redistribution, with soup kitchens relying on cheap, often nutritionally marginal ingredients such as bones, off-cuts, and grains (Carstairs, 2017).

However, the emergence of the Trussell Trust since 1997 fundamentally transformed the CFP field in the UK. By establishing a national network of food banks, formalizing referral-based food parcel distribution, and promoting standardized practices, the Trussell Trust professionalized food support and brought it to national prominence. The organization’s steady growth solidified its role as the dominant actor in the UK CFP field. And while diverse food charities such as soup kitchens, holiday hunger programs, food pantries, and social supermarkets, respond to the same goal, for much of the public, the term “food bank” has become synonymous with the Trussell Trust network. The Trussell Trust experienced significant growth especially after the 2008 Great Recession, particularly following the introduction of the Universal Credit welfare reform a few years later, which intensified demand for food assistance (Reeves & Loopstra, 2021).^6^ Importantly, the Trussell Trust’s model has been historically rooted in mutual solidarity and poverty alleviation, with little emphasis on surplus food redistribution. In fact, its operations rely heavily on public food donations, facilitated through four key mechanisms that never mention food losses or waste.^7^

In contrast, FareShare, another key organization founded in 1994, explicitly centers its mission on environmental sustainability through surplus food redistribution. FareShare is currently the largest surplus food distributor in the UK, and while it also mentions “fighting hunger” in its tagline, its core objective remains reducing food waste. Despite their collaboration—with surplus food from FareShare being redirected to Trussell Trust food banks—the two organizations clearly represent distinct priorities. Notably, FareShare’s operations would persist even if food banks were no longer needed, whereas the Trussell Trust frames its work as temporary, envisioning “a future without food banks.”

This dual emergence, with FareShare focused on food waste mitigation and the Trussell Trust emphasizing poverty relief, has resulted in the development of two interrelated but distinct fields: one focused on the distribution of surplus food and the other focused on distribution of food support (CFP) (Oncini, 2023). The dominance of the Trussell Trust in public discourse has reinforced a framing of food support as primarily a response to poverty rather than a strategy for food waste reduction. This focus has, in turn, given rise to a growing critique of food banks as socially unsustainable (Andriessen et al., 2025; Milbourne, 2024). Independent networks such as IFAN have championed this perspective, advocating for cash-first approaches to address food insecurity at its root rather than perpetuating reliance on food support.^8^

In Japan, the development of CFP followed a markedly different trajectory, heavily influenced by Second Harvest Japan (2HJ), the country’s first formal food bank. Founded in 2002 by Charles McJilton, an American inspired by the US-based Second Harvest (now Feeding America), 2HJ introduced a model that integrates surplus food redistribution and food support, resembling the US-style food banking system. Unlike the UK, where FareShare and the Trussell Trust established distinct roles, 2HJ from the outset combined direct food assistance (e.g., food pantries and soup kitchens) with the redistribution of food stocks to other food charities operating across Japan—and particularly to *kodomo shokudō*.

The role of food banks in Japan was further solidified following the Great East Japan Earthquake in 2011, when 2HJ demonstrated the effectiveness of private organizations in disaster relief by rapidly supplying food to affected regions (Kinoshita & Dollery, 2021; Sano, 2021). This event highlighted the capacity of food banks not only to address chronic food insecurity but also to respond to acute crises, expanding their societal relevance and acceptability to the eyes of the population.

The expansion of food banks in Japan has been both rapid and extensive. Today, the country counts over 280 food banks, most of which are modelled on 2HJ’s integrated approach and prioritize surplus food redistribution. This approach aligns closely with the Japanese cultural ethos of *mottainai*, which emphasizes the moral and practical imperative of avoiding waste (Kimura, 2018; Siniawer, 2014). As a result, Japanese food banks are widely perceived as long-term solutions that simultaneously address hunger and reduce food waste. This complementary framing of social and environmental sustainability is reflected in the slogans and missions of many Japanese food banks, which frequently highlight both objectives in unison.

The contrasting starting points and trajectories of CFP in the UK and Japan have significant implications for how social and environmental sustainability are framed. In the UK, the dominance of poverty relief discourse has positioned food banks as a stopgap measure, with growing critiques of their inability to address systemic food insecurity. This has fueled calls for alternative solutions such as cash-first approaches and, in the words of both Trussell Trust and IFAN, a vision for a future without food banks (Oncini, 2023). Environmental sustainability, while relevant through organizations like FareShare, remains secondary in the broader discourse about poverty relief. Conversely, in Japan, CFP is deeply embedded within a sustainability agenda that integrates social and environmental objectives. Food banks are therefore seen not as temporary fixes but as enduring solutions capable of reducing food waste while addressing hunger. The impetus provided by the 2011 Great East Japan Earthquake likely reinforced this orientation, with social sustainability concerns (namely, ensuring reliable food access in times of crisis) playing a key role in legitimizing and accelerating the development of food charities, while maintaining surplus food redistribution as a core component of their operations. Overall, this comes with an absence of a coordinated critique of food banks’ role, as seen in the UK, which likely reflects the widespread acceptance of their dual role in Japanese society.

### Shared understandings and practices

CFP fields in the UK and Japan share a fundamental purpose: feeding people in need. This shared, taken-for-granted goal drives actors to join existing food charities or establish new ones. In both countries, the volunteer workforce lies at the heart of CFP’s successful functioning. While some of the largest food charities and social enterprises in the UK employ paid staff, the majority of the work is performed by volunteers. These individuals not only provide the labor necessary for food distribution but also embody the spirit of community and mutual aid central to CFP. Despite similar motivations driving participation in CFP, significant differences exist in how the field is internally organized in the two countries. These differences stem partly from the path dependencies outlined earlier and partly from the distinct ways civil society operates within each country.

In the UK, regardless of whether a food charity is affiliated with a larger network, operates independently, or specializes in specific types of food, the CFP field tends to be highly formalized. Informal food support activities are rare, as most food charities operate as formal associations, such as registered charities or social enterprises. The field’s modus operandi is also highly standardized. Most food banks operate within the Trussell Trust network, distributing food parcels through referral mechanisms, often with limits on the number of times individuals can access emergency food. Concurrently, many unaffiliated food support initiatives are part of IFAN, which includes not only food banks but also soup kitchens and food pantries. Food pantries, which operate like grocery stores where members pay a small fee for goods, and soup kitchens, continue to play roles within the field, though their prominence has diminished. Even when churches or sports clubs engage in food support as a secondary activity, their operations typically formalize if expanded or regularized—partly due to the necessity to show compliance with food safety measures.

The UK’s CFP field is further characterized by extensive public discourse on food insecurity and food banks (Knight et al., 2018; Oncini, 2023; Wells & Caraher, 2014). This narrative has shaped public campaigns and fostered a perception of food banks as socially unsustainable—a temporary measure rather than a long-term solution. In fact, national campaigns such as “End Hunger UK” frequently involve collaborations between food charities and media outlets, ensuring that food insecurity remains a prominent issue in public discourse.

In Japan, by contrast, the CFP field is notably fragmented, with significant space for informal activities. While Second Harvest Japan remains the largest and most visible food bank, many other (large or small) food banks operate with similar models focused on managing and redistributing surplus food to organizations and individuals (Nguyen et al., 2026; Nomura, 2020). Beyond the 280 food banks, the field includes a diverse range of actors, such as small local organizations, neighborhood associations (*chōnaikai*), and semi-formal groups. Informality plays a much larger role in Japan’s CFP field, with numerous food support activities organized by community groups or religious organizations without formal registration or oversight. These groups often distribute food parcels or host “food events” on a monthly basis, relying on local promotion methods such as fliers to inform residents.

A distinctive feature of Japan’s CFP field is the widespread presence of *kodomo shokudō*. Initially established in 2012 to provide safe, welcoming spaces for children, particularly those with working parents, *kodomo shokudō* have evolved over time to address broader community needs. Many now focus on supporting low-income families by offering free or low-cost meals for children while also distributing food to households. Their rapid growth underscores their societal importance, with the number of *kodomo shokudō* surpassing 10,000 nationwide as of recent years (Yuasa, 2018). These cafeterias exemplify Japan’s decentralized and community-driven approach to CFP. While some operate as formal organizations, many remain semi-formal or informal, reflecting a broader reliance on grassroots initiatives. Despite growing public awareness of food waste, discussions about the societal factors driving hunger are limited. Food support is often seen as a localized, pragmatic response rather than an avenue for systemic critique or advocacy. This does not mean that such perspectives or advocacy efforts are entirely absent; however, compared to the UK, Japan’s CFP field lacks the level of coordinated campaigns and organized opposition that aims to drive systemic critique and policy change.

Despite organizational differences, food charities in both countries share a common contradiction in how they implement social and environmental sustainability. Surplus food recovery plays a central role in charitable food provision (CFP) in both contexts, yet it consistently falls short of meeting the needs of those seeking assistance. In fact, several of the food charities we interviewed do not rely on surplus food at all. As a result, all food charities in both countries depend heavily on food and monetary donations to operate. These contributions come from government funding, foundation grants, individual donations, fundraising events and partnerships with corporate sponsors, as well as direct donations of new food from producers, manufacturers, and retailers. This reliance challenges the narrative that food insecurity and surplus are naturally complementary. In practice, the social sustainability of CFP fields is only achieved through substantial additional efforts, underscoring the limitations of relying on surplus redistribution alone to address food insecurity.

### The broader field environment

While the broader field environment for CFP includes a wide range of actors, this section focuses on the roles of state actors at both the national and local levels, as well as corporate actors such as producers, manufacturers, and retailers, in shaping the dynamics of the CFP fields in Japan and the UK. These actors play critical roles in influencing the structure, operations, and sustainability of food charities in both countries, albeit in different ways.

In the UK, the role of state actors is far less direct, reflecting the country’s liberal welfare tradition. At the national level, the government’s involvement is primarily regulatory, focusing on setting statutory guidance for the food waste hierarchy. Non-governmental organizations such as WRAP (Waste and Resources Action Programme) are central to promoting food waste reduction, with minimal direct intervention from the government itself. However, beyond providing grants and maintaining the charity register, which tracks the financial accountability of organizations, the government does not attempt to coordinate the CFP. Historically, food banks in the UK even faced scepticism and criticism from some policymakers, with early initiatives framed as unnecessary or even counterproductive (Garthwaite, 2016; Purdam et al., 2016). Over time, however, there has been increasing recognition of the role of food banks in addressing food insecurity. In fact, the UK government now includes references to food banks on its websites, and the House of Commons Library has begun publishing reports on food insecurity (Zaidi, 2024). At the local level, municipalities tend to have a more practical understanding of the needs in their communities. However, rather than directly setting up food banks, local authorities typically facilitate the work of existing food charities, as seen during the COVID-19 pandemic when many municipalities coordinated emergency food distributions (Hirth et al., 2022; Oncini, 2021).

In Japan, state actors are heavily involved in promoting and supporting food banks, particularly at the national level. This involvement is partly motivated by Japan’s extremely low food self-sufficiency ratio, which, at approximately 38%, is one of the lowest among developed countries (Farina, 2017). The Ministry of Agriculture, Forestry, and Fisheries actively views food banks as a dual solution to two critical challenges: reducing food waste and addressing food insecurity. As part of its efforts, the ministry directly supports the development of food banks and integrates them into its broader food security and sustainability strategy. Annual reports provide detailed information on food bank operations, including the number of active organizations, the types of food redistributed, and the overall volumes handled. In addition, it organizes capacity building and networking activities for food banks. This proactive engagement reflects Japan’s developmental state model, which emphasizes close coordination between the state, civil society, and market actors to address societal challenges.

At the local level, Japanese prefectures exercise considerable discretion in managing and supporting food charities. In Kyoto Prefecture, for example, many food charities work closely with the Social Welfare Council, a semi-public institution that bridges government welfare services and community-based initiatives, providing coordination, funding, and support to enhance their operations. These councils often provide food supplies directly to organizations, food banks, and even individuals in need. By maintaining oversight of food support activities within their jurisdiction, local governments also become the means through which the national government keeps track of how CFP is organized.

In both countries however, corporate actors including producers, manufacturers, and retailers, play a critical role in supporting food charities, which shows how social and environmental sustainability are *exploited*. Corporate partnerships provide major food banks in Japan and the UK with essential resources, including surplus food, funding, and sometimes logistical support (e.g., vehicles or venues). These partnerships are often framed as acts of corporate social responsibility, allowing companies to align their brands with social and environmental causes—often without explicit consideration of whether they are donating new food, monetary resources, or facilitating access to surplus food recovery. Food charities, on the other hand, prominently feature corporate logos on their websites and promotional materials, signaling the importance of these partnerships for their operations—especially when carried out with the incumbent actors of the CFP.

### COVID-19 as an exogenous shock

The COVID-19 pandemic acted as a significant exogenous shock to CFP fields in both the UK and Japan, testing their resilience and adaptability under extreme circumstances (Nguyen et al., 2026; Oncini, 2021; Power et al., 2023; Sano, 2021). While the pandemic created common challenges for food charities, the responses and outcomes revealed notable differences in how these systems operate and in the relationship between social and environmental sustainability.

In the UK, the pandemic underscored the friction between food bank operations and the surplus food redistribution system. These two fields, while interconnected, often operate with different priorities and practices, leading to tensions that were amplified during the crisis (Oncini, 2023). Nevertheless, the CFP system in the UK demonstrated considerable resilience and effectiveness, as the pre-existing infrastructure allowed charities to scale up operations rapidly to meet the surge in demand. Despite major logistical and resource challenges, the system’s emergency measures proved resilient, ensuring food aid reached those in need (Oncini, 2023; Power et al., 2023). Despite the higher number of users, however, by 2022 the UK CFP field had largely returned to pre-pandemic modes of operation, with on-site food bank activities resuming. Yet the emergency never fully abated, as ongoing crises such as the cost-of-living crisis and the Russia-Ukraine war have continued to strain the system and perpetuate high levels of food insecurity.

In Japan, the pandemic had a different impact, creating conditions for new actors to emerge and enabling existing organizations to scale up their operations. Many small, informal food charities emerged precisely as a response to the rising levels of food insecurity. This shift was consistently reported by interviewees, who highlighted how the crisis heightened public awareness of food insecurity and brought new resources and attention to the CFP field. Temporary changes in operations, such as social distancing measures and the distribution of meals instead of *in situ* dining, lasted longer in Japan than in the UK. Even in 2023, many organizations continued to adapt their practices, reflecting the broader cautious approach to public health in Japan. This period of sustained adaptation likely allowed Japanese CFP actors to strengthen their infrastructure and expand their reach, particularly through grassroots initiatives like *kodomo shokudō*, which became even more prominent during the crisis.

In both countries, the pandemic prompted temporary operational changes, but it also highlighted the enduring differences in social and environmental sustainability. In the UK, the focus remained heavily on addressing immediate food insecurity, with little integration of environmental goals into the broader narrative of CFP. While food waste reduction is a stated goal of the UK government, this dimension is primarily emphasized in environmental policies rather than as part of a comprehensive approach to food charity operations. In Japan, by contrast, the pandemic reinforced the complementarity between social and environmental sustainability, with increased emphasis on redistributing surplus food to initiatives like *kodomo shokudō*.

## Discussion and conclusions

Building on the SAF theoretical approach, this paper has sketched a comparative analysis of CFP in Greater Manchester and Kyoto Prefecture, focusing on the consequences that differing field organizational models have for social and environmental sustainability. In both contexts, CFP has become an indispensable safety net for individuals facing poverty, evolving into highly professionalized systems with extensive reach. Despite their contextual differences, CFP fields in both regions reflect the broader trends and tensions shaping food support systems globally, particularly concerning their alignment with sustainability goals and the transition towards more just food systems.

The analysis underscores how the historical trajectories and foundational logics of CFP fields shape their current operations and sustainability framing. In the UK, the dual emergence of the Trussell Trust and FareShare has created two interrelated but distinct fields: one focused on addressing food insecurity and the other on surplus food redistribution. This bifurcation has positioned poverty alleviation as the primary framing for CFP in the UK, with the environmental consideration somewhat detached from it. In Japan, by contrast, the integration of surplus food redistribution and food support from the outset has fostered a framing in which the complementarity of reducing food waste and addressing hunger is taken for granted. These differences highlight how organizational models and historical path dependencies shape the articulation and prioritization of sustainability goals within CFP fields.

Operational practices further reveal contrasts between the two contexts. In the UK, CFP is characterized by formalized, network-based organizations, with structured processes such as referrals and partnerships dominating the landscape. In Japan, CFP is more decentralized and community-driven, with grassroots initiatives like *kodomo shokudō* and informal food distribution events reflecting a localized and participatory approach. These distinctions are deeply rooted in the broader socio-political and cultural environments of each country, with the UK’s liberal welfare tradition favoring formalization and Japan’s developmental state model emphasizing community engagement and coordination.

Despite these differences, achieving full complementarity between social and environmental sustainability remains a challenge in both contexts. While surplus food redistribution is central to CFP, it is insufficient to meet the needs of all those requiring assistance. Food charities universally rely on additional donations, whether monetary or newly sourced food, to supplement the surplus food they receive. This reliance is in continuation with a model rooted more in traditional charitable giving, which evidently continues to play a fundamental role in (food) poverty alleviation efforts.

Furthermore, the involvement of corporate actors in both countries reflects similar dynamics. Corporate partnerships provide essential resources for food charities, yet they also embed CFP within a framework of corporate social responsibility that prioritizes reputational benefits. This dynamic reinforces the systemic reliance on corporate support, even as it highlights the tension between short-term operational needs and the broader goal of transforming unsustainable food systems.

In contributing to the sustainability literature, this paper illuminates the complex and often ambiguous relationship between social and environmental sustainability. While the “win–win” narrative (Ariztia & Araneda, 2022) portrays CFP as simultaneously addressing food insecurity and food waste, the findings suggest that this complementarity remains largely aspirational in both contexts. Moreover, the paper shows that the relationship between these two pillars of sustainability is shaped by the organization and historical evolution of CFP fields, and thus varies across settings. This underscores the value of comparative field analysis in revealing how cultural and socioeconomic factors influence the articulation—and the limitations—of sustainability goals, as well as the challenges of developing shared frameworks. In doing so, the paper also contributes to broader debates on the inequality–unsustainability nexus (Darmon, 2024), demonstrating how these interlinked crises are embedded differently across institutional and geographic contexts. By showing how food support systems mediate these tensions in distinctive ways, the analysis affirms the value of comparative research not only for understanding CFP, but also for uncovering field-specific entanglements in other domains—such as housing, energy, or education—where inequality and (un)sustainability similarly intersect, though in diverse configurations.

SAF theory has proven to be a promising tool for comparative analysis, particularly given the widespread emergence of CFP systems in high-income and, increasingly, in middle-income countries over the past two decades. While these systems share structural similarities, they are embedded in diverse civil society regimes, welfare traditions, and food markets. Future research should delve deeper into these contextual differences to better understand how CFP fields operate across various settings. Comparative studies of other urban areas could provide valuable insights into the interplay of social and environmental sustainability, revealing both commonalities and divergences in how food support systems function globally.

Nevertheless, a critical dimension absent from this paper is the perspective of those who actually rely on food support. Understanding how individuals navigate, and experience CFP fields is essential to comprehending how these systems shape—and are shaped by—the survival strategies of those in need. Participant observation and ethnographic research conducted over extended periods will be indispensable for capturing these lived experiences, offering a more comprehensive view of the role of CFP in addressing food insecurity and its broader societal implications. Furthermore, fostering collaboration and information exchange among all actors involved in the functioning of food support, from both the provision and the usage side, will be fundamental for gaining a more comprehensive understanding of the current trajectory of CFP, its potential improvements, and the pathways toward wider changes in the food system.

This paper thus aims to lay the groundwork for further research into the systemic patterns and contextual variations within and between CFP fields. By doing so, it advocates for the development of a more inclusive and participatory research agenda. Such an agenda is not only necessary for deepening our understanding of CFP but also for informing policies and practices that advance both social and environmental sustainability in meaningful and transformative ways.

## Data Availability

The data supporting the findings of this study cannot be shared publicly due to ethical considerations and confidentiality agreements made with participants. As outlined in the ethics approval for this research, participants were assured that their information would remain confidential and would not be disclosed or shared with third parties. This commitment was integral to obtaining informed consent and ensuring the privacy and trust of participants throughout the study.

## Footnotes

1 SDG 1 (No Poverty) emphasizes poverty eradication, including improving access to resources such as food for vulnerable populations. SDG 2 (Zero Hunger) focuses on ending hunger and malnutrition while ensuring food security. SDG 12 (Responsible Consumption and Production) highlights the importance of reducing food waste and promoting sustainable resource use.

2 For instance, the food waste hierarchy (Papargyropoulou et al., 2014) suggests food waste prevention as the most effective measure, followed by the redistribution of surplus food to those in need as the second most effective one.

3 This paper does not consider “economic sustainability” but rather focuses on the tensions between environmental and social sustainability for two reasons. First, the Brundtland Report’s framing of economic sustainability relies on a disembedded view of the economy, treating it as separate from society—a perspective that becomes evident in its focus on economic growth as a standalone objective. Second, from a relational perspective, such disentanglement between the social and the economic is unnecessary, as economic actions are embedded within social systems (Granovetter, 1985), thus implying that economic sustainability cannot be meaningfully disentangled from social sustainability.

4 Further clarifications on *kodomo shokudō* follow later in the text.

5 GMPA is still active, but now operates under the name Resolve Poverty.

6 Universal Credit is a major UK welfare reform introduced in 2013 and rolled out nationally from 2014. It consolidates six means-tested benefits, into a single monthly payment. It extends conditionality and sanctions, is paid in arrears, and is administered primarily through an online system, with the stated aim of simplifying the benefit system and incentivizing employment. However, it has been widely criticized for increasing financial hardship, particularly due to payment delays, benefit reductions, and difficulties in navigating the digital system (Reeves and Loopstra, 2021).

7 Drop-off points in supermarkets across the country; Financial contributions to local food banks for targeted purchasing; Online food donations through supermarket delivery platforms; Community-based food collection drives (e.g., schools, churches, businesses).

8 See https://www.foodaidnetwork.org.uk/cash-first. Accessed 08/10/2025.
